# Reactive Carbonyl Species Mediate Isothiocyanate Signaling Pathway in *Arabidopsis thaliana* Guard Cells

**DOI:** 10.1111/ppl.70775

**Published:** 2026-02-11

**Authors:** Sumaiya Farzana, Md. Moshiul Islam, Toshiyuki Nakamura, Yoshimasa Nakamura, Shintaro Munemasa, Jun'ichi Mano, Yoshiyuki Murata

**Affiliations:** ^1^ Graduate School of Environmental and Life Science Okayama University Okayama Japan; ^2^ Department of Agronomy Gazipur Agricultural University Gazipur Bangladesh; ^3^ Science Research Center Yamaguchi University Yamaguchi Japan

**Keywords:** arabidopsis, GSH depletion, isothiocyanate, reactive carbonyl species, reactive oxygen species

## Abstract

Our previous results demonstrated that depletion of glutathione (GSH) rather than elevation of levels of reactive oxygen species (ROS) is highly correlated with the decrease in stomatal aperture induced by isothiocyanates (ITCs), although ROS is considered a key second messenger in stomatal closure, suggesting that another signal component regulates stomatal apertures along with GSH depletion. This study, using Arabidopsis, clarified that reactive carbonyl species (RCS), especially acrolein and 4‐hydroxy‐(*E*)‐2‐nonenal, are determinants of stomatal aperture responses to ITCs. All tested ITCs, allyl isothiocyanate (AITC), sulforaphane (SFN), benzyl isothiocyanate (BITC), and phenethyl isothiocyanate (PEITC), significantly induced stomatal closure, which was inhibited by the RCS scavengers, carnosine and pyridoxamine. The RCS scavengers suppressed ITC‐induced depletion of GSH but not elevation of ROS levels. All tested ITCs (AITC, SFN, BITC, and PEITC) increased levels of RCS and non‐RCS aldehydes in the epidermal tissues. However, acrolein, 4‐hydroxy‐(*E*)‐2‐nonenal, crotonaldehyde, and (*E*)‐2‐pentenal induced stomatal closure at 10 and 100 μM, whereas propionaldehyde, butyraldehyde, and *n*‐pentanal did not at concentrations up to 100 μM. Acrolein and 4‐hydroxy‐(*E*)‐2‐nonenal more effectively induced stomatal closure and GSH depletion than crotonaldehyde and (*E*)‐2‐pentenal did. The contents of RCS were more strongly correlated with GSH levels and stomatal closure than with ROS levels. These results suggest that RCS, especially acrolein and 4‐hydroxy‐(*E*)‐2‐nonenal, acts as key regulators of stomatal closure in guard cells in response to ITCs.

## 1 Introduction

Stomata are composed of a pair of guard cells, which are present in the epidermis of higher plants. Stomata facilitate gas exchange, regulate water loss, and defend against microbial invasions through their opening and closing mechanisms. Guard cells are influenced by various biotic and abiotic stresses, including drought, CO_2_ levels, light, humidity, and plant hormones such as abscisic acid and methyl jasmonate (Melotto et al. 2017; Shimazaki et al. 2007; Murata et al. 2015; Ye et al. 2015). Secondary metabolites such as isothiocyanates (ITCs) play a significant role in stomatal movement (Bednarek 2012).

Plants in the family Brassicaceae, including *Arabidopsis thaliana*, possess the glucosinolate‐myrosinase system (Ishida et al. 2014). Glucosinolate hydrolysis is limited in intact tissues because glucosinolates and myrosinases are separately localized. However, in plants that have suffered mechanical damage and herbivore attack, ITCs are produced through the degradation of glucosinolates by myrosinase (Bones and Rossiter 1996). In ground leaves of Arabidopsis, ITC levels have been estimated to be approximately 10 μM g^−1^ FW (fresh weight; Khokon et al. 2011) and approximately 2 mg g^−1^ FW in cabbage sprouts (Wang et al. 2015), which indicates that ITC content can reach around 1 mM in ground leaves and up to 10 μM in certain damaged parts of leaves. Moreover, guard cells occupy less than 10% (v/v) of the epidermal tissues (Islam et al. 2019). Hence, it is possible for ITC concentration to regionally reach 50 μM, which was employed in this study.

ITCs are highly reactive because of their electrophilicity (Zhang et al. 1995). ITCs have effects on a plant's various physiological processes. ITCs inhibit growth in multiple plant species, including *A. thaliana* (Norsworthy and Meehan 2005; Hara et al. 2010). Several ITCs, allyl isothiocyanate (AITC), sulforaphane (SFN), benzyl isothiocyanate (BITC), and phenethyl isothiocyanate (PEITC), triggered the elevation of reactive oxygen species (ROS) levels and depletion of intracellular glutathione (GSH), resulting in stomatal closure both in glucosinolate‐producing plants such as Arabidopsis (Afrin et al. 2020; Khokon et al. 2011) and non‐glucosinolate‐producing plants such as *Vicia faba* (Sobahan et al. 2015). We chose four ITCs (AITC, SFN, BITC, and PEITC) that differ in their reactivity, electron density, and hydrophobicity (Afrin et al. 2020; Aihara et al. 2023). The AITC‐induced elevation of ROS levels is mediated by salicylhydroxamic acid‐sensitive peroxidases in *A. thaliana* (Hossain et al. 2013) and *V. faba* (Sobahan et al. 2015). However, the downstream events triggered by ROS in the guard‐cell ITC signaling pathway remain unclear. Furthermore, the elevation of ROS levels by ITCs is not so highly correlated with the decrease in stomatal aperture induced by ITCs as GSH depletion, although ROS is considered a key second messenger in stomatal closure (Afrin et al. 2020). Hence, it remains to be clarified what signal component dominantly regulates stomatal apertures during ITC‐induced stomatal closure.

ROS are constitutively produced in plant cells and can oxidize membrane lipids, especially polyunsaturated fatty acids, into lipid peroxides. Lipid peroxides can break down into carbonyl compounds, such as aldehydes and ketones, through either enzymatic or non‐enzymatic processes (Mueller 2004; Mano 2012). Carbonyl compounds with *α,β*‐unsaturated bonds, such as acrolein and 4‐hydroxy‐(*E*)‐2‐nonenal (HNE), are grouped as reactive carbonyl species (RCS) (Mano 2012), which are more electrophilic than simple aldehydes and ketones. The excessive RCS is produced in plants under environmental stress, such as strong light (Mano et al. 2010), high temperature (Kai et al. 2012), and salt stress (Sultana et al. 2022), and can cause damage to plant cells. However, RCS also functions as a signaling molecule downstream of ROS production in guard‐cell hormone signaling and chitosan signaling at lower concentrations, leading to stomatal closure (Islam et al. 2016, 2019, 2020; Jahan et al. 2025). The concentrations of RCS were 1 to 10 nmol g^−1^ FW in tobacco leaves (Mano et al. 2010) and from 0.1 to 12 nmol g^−1^ FW in Arabidopsis leaves (Mano et al. 2014), which are in the order of micromolar. Furthermore, epidermal tissues contain guard cells and other epidermal cells; however, ROS production upstream of RCS production was observed only in guard cells, not in the other epidermal cells (Islam et al. 2019). This study employed epidermal tissues to measure RCS contents. Guard cells occupy less than 10% (v/v) of the epidermal tissues, and the reaction of RCS production occurs in the vicinity of the membrane (Islam et al. 2019). Hence, it is possible for RCS concentration to regionally reach 100 μM. The RCS scavengers, carnosine and pyridoxamine, suppressed RCS accumulation but not ROS accumulation in H_2_O_2_‐treated tobacco BY‐2 cells and abscisic acid‐treated Arabidopsis and tobacco epidermal tissues (Biswas and Mano 2016; Islam et al. 2016, 2019). However, it remains to be clarified whether RCS are involved in ITC‐induced stomatal closure and what RCS dominantly induces stomatal closure.

GSH is essential for redox signaling, detoxification, and compound metabolism in plants (Ito and Ohkama‐Ohtsu 2023) and influences various hormone signaling pathways (Noctor et al. 2012). GSH negatively regulates guard‐cell ITC signaling that leads to stomatal closure in *A. thaliana* (Afrin et al. 2020; Khokon et al. 2011) and in *V. faba* (Sobahan et al. 2015), as well as guard‐cell abscisic acid, methyl jasmonate, salicylic acid, and chitosan signaling in *A. thaliana* (Okuma et al. 2011; Akter et al. 2013, 2022; Jahan et al. 2024).

This study investigated RCS production in response to ITCs (AITC, SFN, BITC, and PEITC) and the effects of RCS scavengers, carnosine and pyridoxamine, on ITC‐induced stomatal movement, elevation of ROS levels, and GSH content to clarify the involvement of RCS in the guard‐cell ITC signaling pathway. Furthermore, we examined stomatal response to several aldehydes and ketones that were produced during ITC‐induced stomatal closure to determine whether RCS is a regulatory intermediate in the guard‐cell ITC signaling pathway.

## 2 Materials and Methods

### 2.1 Plant Materials and Growth Conditions

Wild‐type *Arabidopsis thaliana* Columbia‐0 (Col‐0) was grown in a plastic pot with a soil mixture of 70% vermiculite (Asahi‐Kogyo) and 30% (v/v) soil (Kureha Chemical) in a growth chamber at 21°C ± 2°C. The plants were maintained under a 16/8‐h light/dark cycle with a light intensity of 80 μmol m^−2^ s^−1^. Plants were watered weekly with distilled water containing 0.1% Hyponex (Hyponex).

### 2.2 Measurement of Stomatal Aperture

Stomatal aperture measurements were conducted using the method outlined by Afrin et al. (2020). Rosette leaves from 4‐ to 6‐week‐old plants were selected, excised, and placed in a stomatal bioassay solution containing 5 mM KCl, 50 μM CaCl_2_, and 10 mM 2‐(*N*‐morpholino)ethanesulfonic acid‐tris(hydroxymethyl)aminomethane (pH 5.6) for 2 h to induce stomatal opening. Then, 0.1% dimethyl sulfoxide or 50 μM AITC (Tokyo Chemical Industry [TCI], purity > 95.0%, specific gravity = 1.02), SFN (Sigma Aldrich), BITC (TCI, purity > 97.0%, specific gravity = 1.21), or PEITC (TCI, purity > 97.0%, specific gravity = 1.11) (all were dissolved in dimethyl sulfoxide) or acrolein (TCI, purity > 97.0%, specific gravity = 0.85) or HNE (Enzo Life Science) or crotonaldehyde (TCI, purity > 98.0%, specific gravity = 0.85) or (*E*)‐2‐pentenal (TCI, purity > 95.0%, specific gravity = 0.85) or propionaldehyde (TCI, purity > 98.0%, specific gravity = 0.81) or butyraldehyde (TCI, purity > 98.0%, specific gravity = 0.81) or *n*‐pentanal (TCI, purity > 95.0%, specific gravity = 0.82) was added to the solution for another 2 h incubation under light condition. Scavengers of RCS, carnosine (1 mM), and pyridoxamine (0.5 mM), were applied 30 min before the ITCs application. After blending the incubated leaves in a commercial blender, the epidermal tissues were collected using a nylon mesh (pore size: 100 μm). From three independent experiments, at least 20 stomatal apertures were measured from two leaves collected from two independent plants using WinRoof 3.0 software (Mitani Corporation).

According to Afrin et al. (2020), we subtracted “stomatal aperture of ITCs‐treated leaves (μm)” from “stomatal aperture of dimethyl sulfoxide‐treated leaves (μm)” to obtain “decrease in stomatal aperture (μm)”.

### 2.3 Measurement of ROS Levels in Guard Cells

ROS levels in guard cells were measured using 2′,7′‐dichlorodihydrofluorescein diacetate (Sigma) as previously explained (Afrin et al. 2020). Leaves were blended in a commercial blender, and epidermal tissues were collected by nylon mesh (pore size: 100 μm). The blended epidermal tissues were used for measuring ROS levels due to the interference of chlorophyll fluorescence during observation and the elevation of ROS levels triggered by blending. The epidermal tissues were soaked in a stomatal bioassay solution containing 5 mM KCl, 50 μM CaCl_2_, and 10 mM 2‐(*N*‐morpholino)ethanesulfonic acid‐tris(hydroxymethyl)aminomethane (pH 5.6) under light for 2 h before treatment application to avoid effects of blending on ROS levels. The tissues were treated with 50 μM 2′,7′‐dichlorodihydrofluorescein diacetate under dark conditions at 25°C ± 1°C for 30 min. The epidermal tissues were collected after washing away excess dye and then treated with 0.1% dimethyl sulfoxide and 50 μM ITCs (AITC, SFN, BITC, or PEITC) in the dark at 25°C ± 1°C for an additional 30 min. The application of ITCs elicited an elevation of ROS levels in the guard cells within 15 min, and the ROS level reached a maximum within 30 min and then gradually decreased (Figure S2), a response similar to that induced by abscisic acid and methyl jasmonate (Suhita et al. 2004). Hence, ROS levels were measured 30 min after ITC treatment. For each experiment, four or five leaves were collected from separate plants, and 20 guard cells were examined from fluorescence images for each replication (*n* = 3). Then, a fluorescence microscope (Biozero BZ‐8000, KEYENCE) with an OP‐66835 BZ filter was used to observe the fluorescence of the guard cells (excitation wavelength: 480 nm; emission wavelength: 510 nm). The fluorescence intensity of images was analyzed using ImageJ 1.42q software (National Institutes of Health [NIH], Bethesda, MD).

### 2.4 Measurement of GSH Content in Guard Cells

GSH levels in guard cells were quantified fluorometrically using monochlorobimane, following the method described by Okuma et al. (2011). The leaves were shredded using the blender, and tissues were collected with a nylon mesh (pore size: 100 μm). The blended epidermal tissues were employed for GSH content measurement because blending may decrease GSH content. The epidermal tissues were placed in a stomatal bioassay solution containing 5 mM KCl, 50 μM CaCl_2_, and 10 mM 2‐(*N*‐morpholino)ethanesulfonic acid‐tris(hydroxymethyl)aminomethane (pH 5.6) under light for 2 h of incubation to avoid blending effects on GSH contents. The epidermal tissues were treated with 0.1% dimethyl sulfoxide, or 50 μM AITC, SFN, BITC, or PEITC, or acrolein, HNE, crotonaldehyde, (*E*)‐2‐pentenal, propionaldehyde, butyraldehyde, or *n*‐pentanal in the presence of monochlorobimane for 2 h at 25°C ± 1°C. The amounts of GSH in guard cells gradually decreased after ITC application and reached a minimum within 2 h, and the stomatal apertures also decreased with time and reached a minimum 2 h after ITC application (Figure S3), which is similar to abscisic acid‐induced depletion of GSH (Okuma et al. 2011). Therefore, GSH amounts were measured 2 h after ITC treatment. Three independent experiments were conducted. The RCS scavengers (carnosine at 1 mM; pyridoxamine at 0.5 mM) were applied to the sample 30 min prior to the addition of ITCs, RCS, and monochlorobimane. Guard cell fluorescence was observed using a fluorescent microscope (Biozero BZ‐8000, KEYENCE) with an OP‐66834 BZ filter (excitation wavelength: 360 nm; emission wavelength: 460 nm). The ImageJ 1.42q software (NIH) was used to measure the fluorescence intensity of images. Five or six leaves were collected from five or six independent plants, and a minimum of 20 guard cells were analyzed from the fluorescence images. Note that application of GSH increases fluorescence through conjugate formation with monochlorobimane (Rice et al. 1986), but application of RCS (acrolein) or ITC (AITC) does not significantly change fluorescence (Figure S4), which is consistent with the chemical properties of these compounds: nucleophilicity of GSH and electrophilicities of RCS and ITC.

### 2.5 Identification and Quantification of RCS

Arabidopsis rosette leaves from 4‐ to 6‐week‐old plants were excised and blended for 30 s. Epidermal tissues were collected by nylon mesh (pore size: 100 μm) and soaked in a bioassay solution containing 5 mM KCl, 50 μM CaCl_2_, and 10 mM 2‐(*N*‐morpholino)ethanesulfonic acid‐tris(hydroxymethyl)aminomethane (pH 5.6) for 2 h in the light, followed by the addition of 50 μM of ITCs (AITC, SFN, BITC, or PEITC) for 40 min. From 1 g of epidermal tissues, RCS was extracted, derivatized with 2,4‐dinitrophenylhydrazine, and analyzed using reverse‐phase high‐performance liquid chromatography following the described procedure (Islam et al. 2016). Dinitrophenylhydrazone derivatives of RCS were identified based on their retention times and quantified by comparing them with authentic standards (Matsui et al. 2009). The baselines drifted by less than 0.001 (Figure 4A–D), which may be due to fluctuations in temperature. The difference in extraction efficiencies was within 93% ± 2% in this experiment.

### 2.6 Determination of the Partition‐Coefficient (Log *P*) Values of RCS

Partition‐coefficient (log *P*) values were calculated using ChemDraw software (version 22.2.0, 32‐bit, PerkinElmer, 2022).

### 2.7 Statistical Analysis

Statistical analysis was performed using Statistical Tools for Agriculture Research (STAR) 2.0.1 (2014), International Rice Research Institute (Manila, Philippines). One‐way analysis of variance (ANOVA) followed by Tukey's test (*p* < 0.05) and Student's *t*‐test (*p* < 0.05) were used to assess treatment differences. Correlation analysis was conducted in Microsoft Excel.

## 3 Results

### 3.1 Effects of RCS Scavengers on Stomatal Responses to ITCs

**FIGURE 1:**
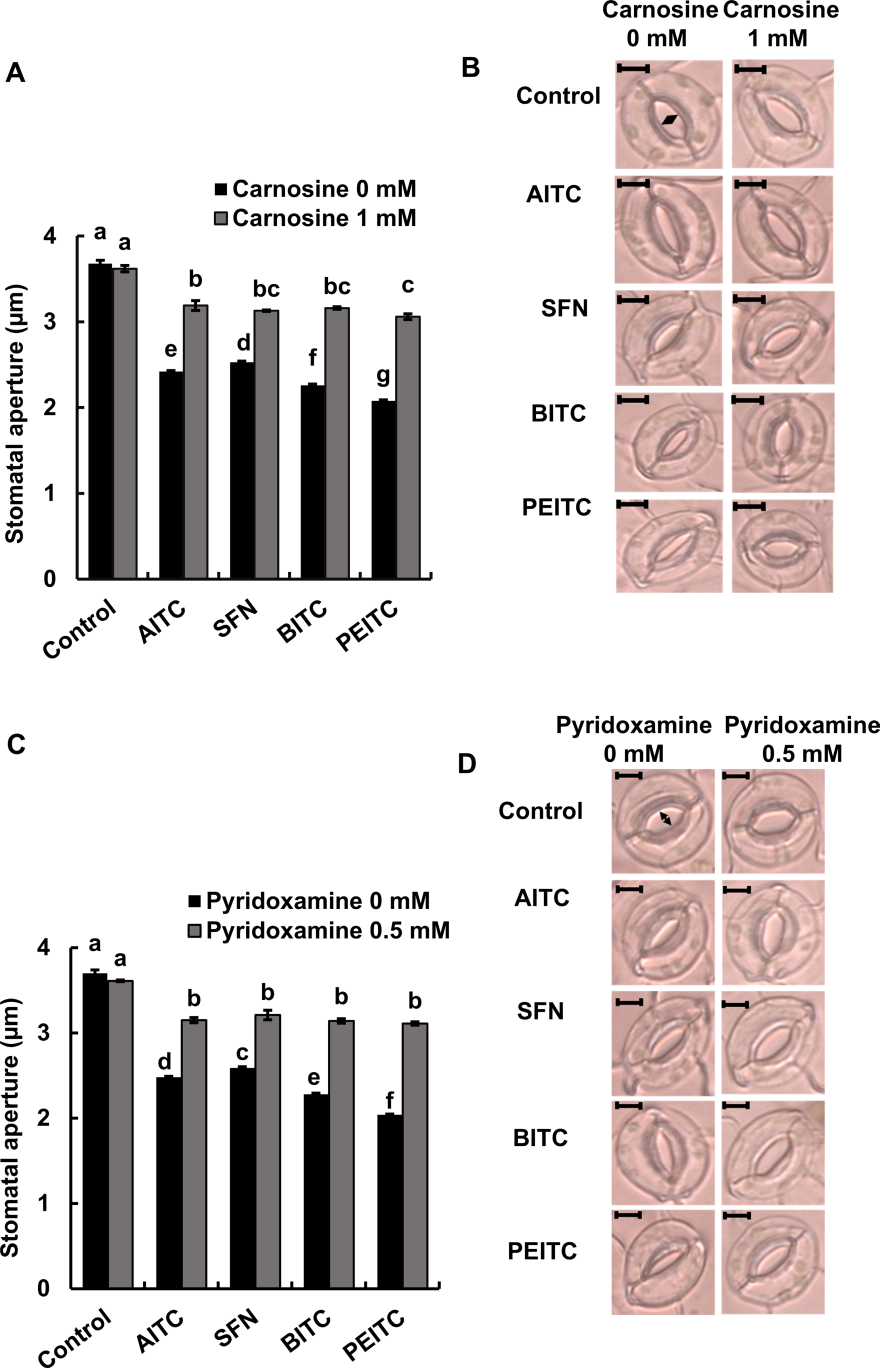
Effects of RCS scavengers on ITC‐induced stomatal closure. Arabidopsis rosette leaves were treated with 50 μM allyl isothiocyanate (AITC), sulforaphane (SFN), benzyl isothiocyanate (BITC), and phenethyl isothiocyanate (PEITC) for 2 h after pretreated with RCS scavengers, carnosine (A) and pyridoxamine (C), for 30 min. Each bar is presented as the mean of three replications (60 stomata per bar) ± standard error of the mean. Different letters indicate statistical significance (*p <* 0.05, with Tukey's test). The images show Arabidopsis guard cells treated with ITCs in the absence and presence of carnosine (B) or pyridoxamine (D). Scale bar: 5 μm.

To investigate whether RCS are involved in the guard‐cell ITC signaling in Arabidopsis, we measured ITC‐induced stomatal apertures using the RCS scavengers, carnosine and pyridoxamine. Both carnosine at 1 mM (Figure 1A,B) and pyridoxamine at 0.5 mM (Figure 1C,D) significantly inhibited the ITC‐induced stomatal closure (*p* < 0.05, Tukey's test) but not completely. When the epidermal tissues were employed, similar results were also obtained (Figure S1).

**FIGURE 2:**
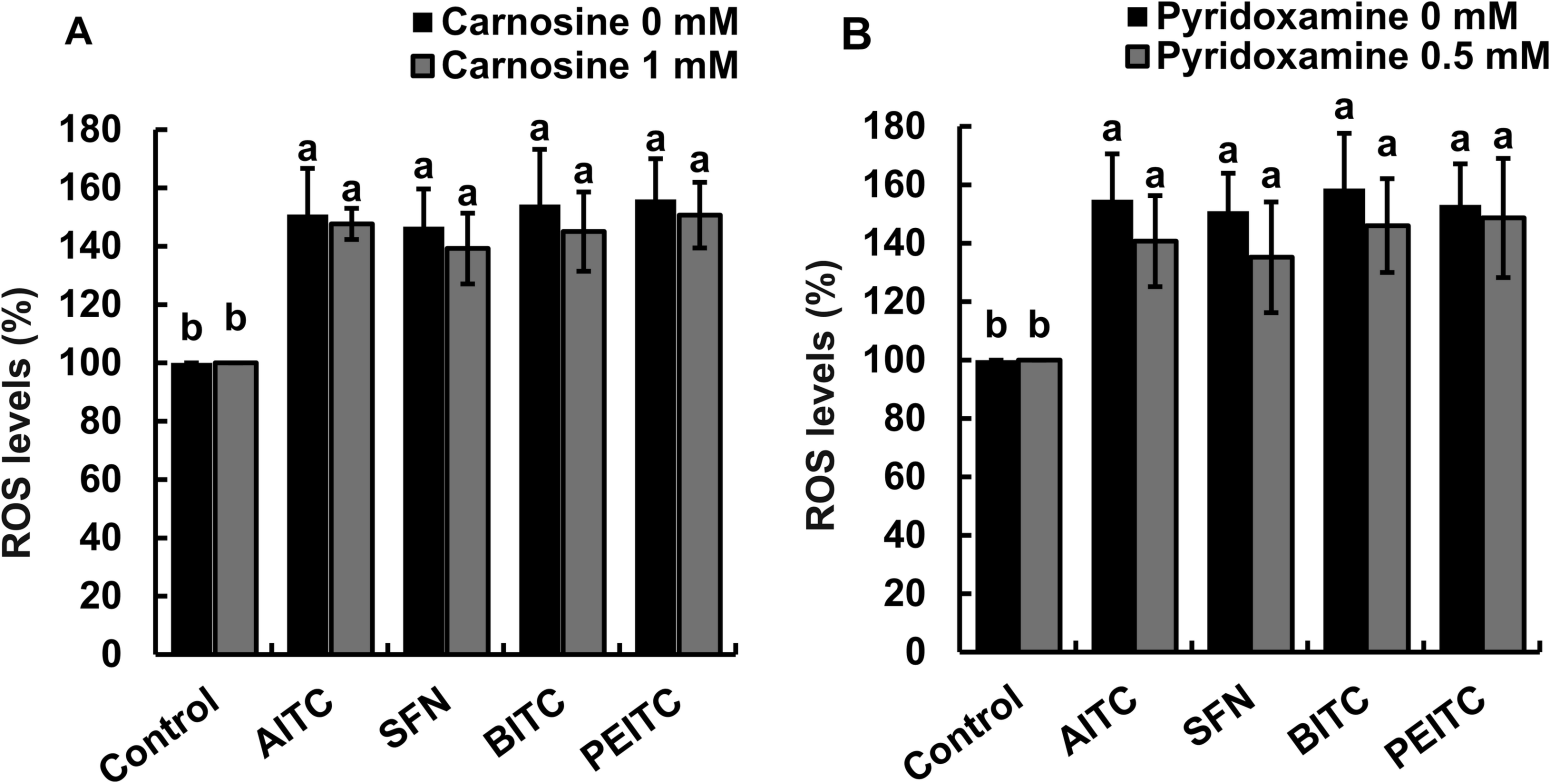
Effects of RCS scavengers on ITC‐induced elevation of ROS levels in guard cells. Epidermal tissues were incubated with 2′,7′‐dichlorodihydrofluorescein diacetate for 30 min and then were treated with 50 μM allyl isothiocyanate (AITC), sulforaphane (SFN), benzyl isothiocyanate (BITC), and phenethyl isothiocyanate (PEITC) in the absence or presence of carnosine (A) or pyridoxamine (B). Dimethyl sulfoxide (0.1%, control) does not affect ROS levels. Samples were pretreated with RCS scavengers for 30 min before ITCs application. The vertical axis shows the percentage of ROS levels. Each bar shows the average data of 60 guard cells (*n* = 3) ± standard error of the mean. Different letters indicate statistical significance (*p <* 0.05, with Tukey's test).

To clarify that RCS production occurs downstream of ROS, we investigated the effects of RCS scavengers on ROS induced by ITCs in guard cells. Neither carnosine (Figure 2A) nor pyridoxamine (Figure 2B) inhibited the elevation of ROS levels by ITCs in guard cells, although ITCs, AITC, BITC, SFN, and PEITC significantly elevated ROS levels in guard cells (Figure 2).

**FIGURE 3:**
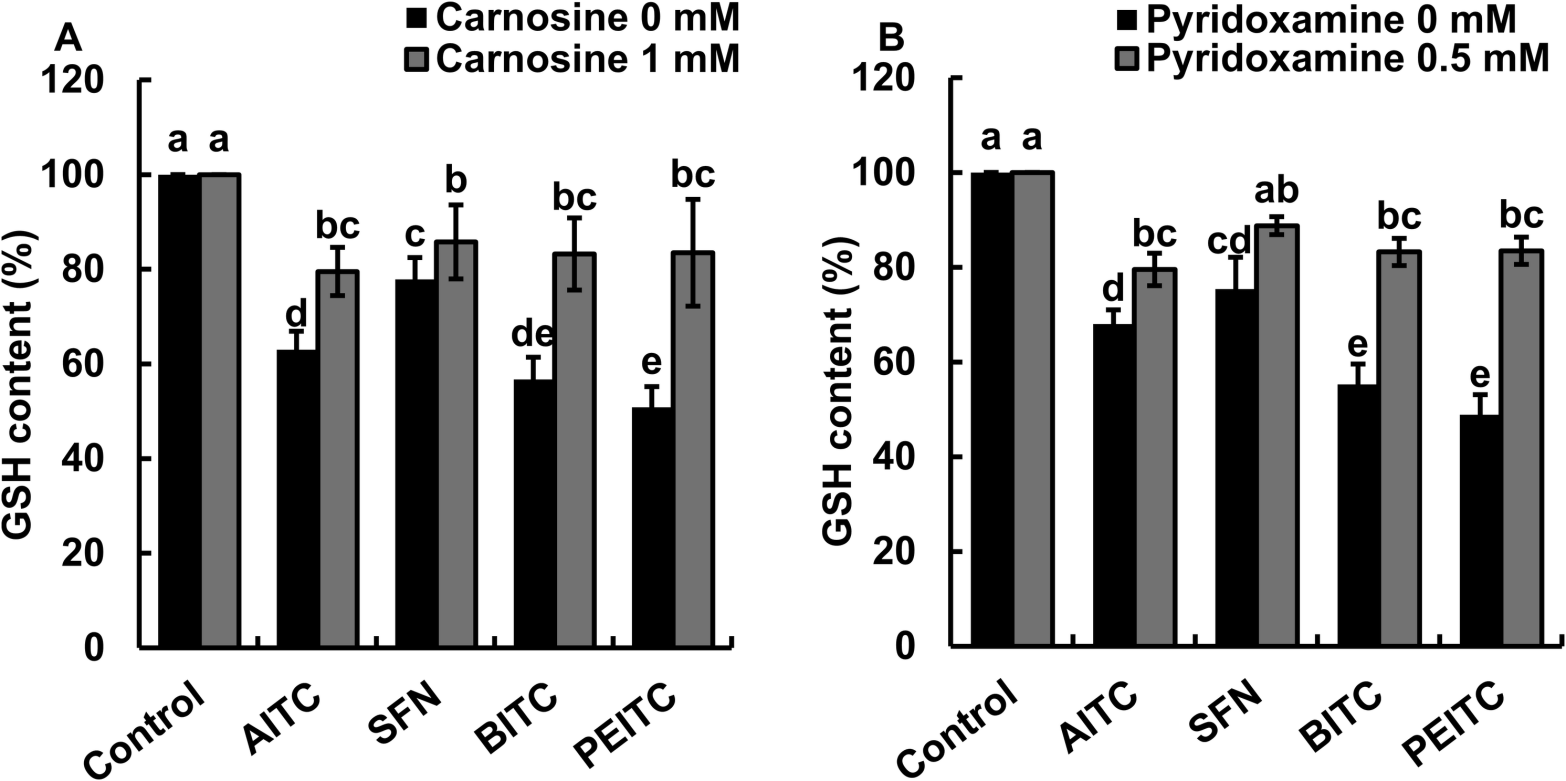
Effects of RCS scavengers on ITC‐induced GSH content in guard cells. Epidermal tissues were treated with 50 μM allyl isothiocyanate (AITC), sulforaphane (SFN), benzyl isothiocyanate (BITC), and phenethyl isothiocyanate (PEITC) in the presence of monochlorobimane for 2 h after pretreated with RCS scavengers, carnosine (A) and pyridoxamine (B), for 30 min. Dimethyl sulfoxide (0.1%, control) as a solvent does not affect GSH content in guard cells. The vertical axis shows the percentage of GSH content. Each bar shows the average data of 60 guard cells (*n* = 3) ± standard error of the mean. Different letters indicate statistical significance (*p <* 0.05, with Tukey's test).

To determine the involvement of RCS in ITC‐induced GSH depletion, we measured GSH contents in guard cells in the absence and presence of the RCS scavengers (Figure 3). Both 1 mM carnosine (Figure 3A) and 0.5 mM pyridoxamine (Figure 3B) pretreatments significantly inhibited the ITC‐induced GSH depletion in guard cells (*p* < 0.05, Tukey's test), while GSH levels in guard cells were not recovered to control levels except for the GSH level of SFN‐treated guard cells in the presence of pyridoxamine.

### 3.2 ITC‐Induced RCS Production

**FIGURE 4:**
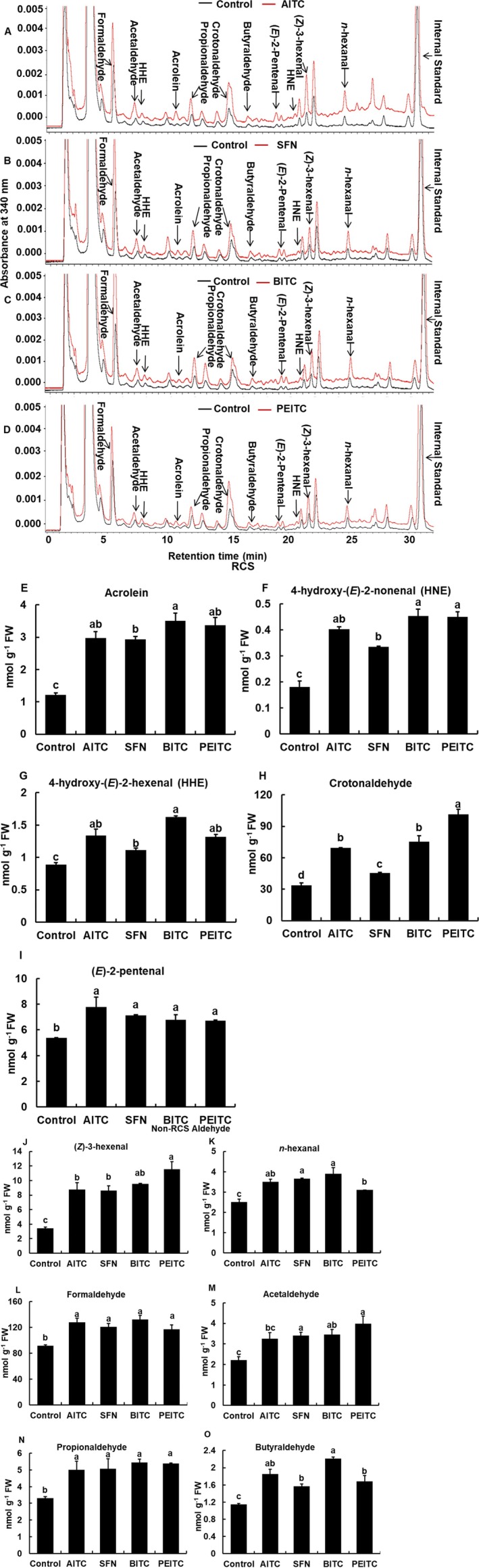
Carbonyl contents in the epidermal tissues of Arabidopsis treated with ITCs. (A–D) Chromatograms of the dinitrophenylhydrazone derivatives of carbonyls extracted from control (black lines) and ITC‐treated (red lines) epidermal tissues of Arabidopsis. Carbonyls are marked above each peak. (E–I) Contents of RCS and (J–O) non‐RCS aldehydes in the epidermal tissues of Arabidopsis in response to ITCs (AITC, SFN, BITC, and PEITC). Epidermal tissues were incubated for 2 h under light conditions and then treated with 50 μM ITCs for 40 min. Error bar represents the standard error of the mean (*n* = 3). Different letters indicate statistical significance (*p <* 0.05, with Tukey's test).

We measured RCS contents in the epidermal tissues of Arabidopsis rosette leaves treated with 50 μM ITCs (AITC, SFN, BITC, and PEITC). Treatment with 50 μM ITCs significantly (*p* < 0.05, Tukey's test) increased acrolein (Figure 4A–E), HNE (Figure 4A–D,F), 4‐hydroxy‐(*E*)‐2‐hexenal (HHE) (Figure 4A–D,G), crotonaldehyde (Figure 4A–D,H), and (*E*)‐2‐pentenal (Figure 4A–D,I). Application of ITCs also significantly increased (*Z*)‐3‐hexenal (Figure 4A–D,J), *n*‐hexanal (Figure 4A–D,K), formaldehyde (Figure 4A–D,L), acetaldehyde (Figure 4A–D,M), propionaldehyde (Figure 4A–D,N), and butyraldehyde (Figure 4A–D,O), which are non‐RCS aldehydes.

### 3.3 Stomatal Closure and GSH Depletion Induced by RCS and Non‐RCS Aldehyde

**FIGURE 5:**
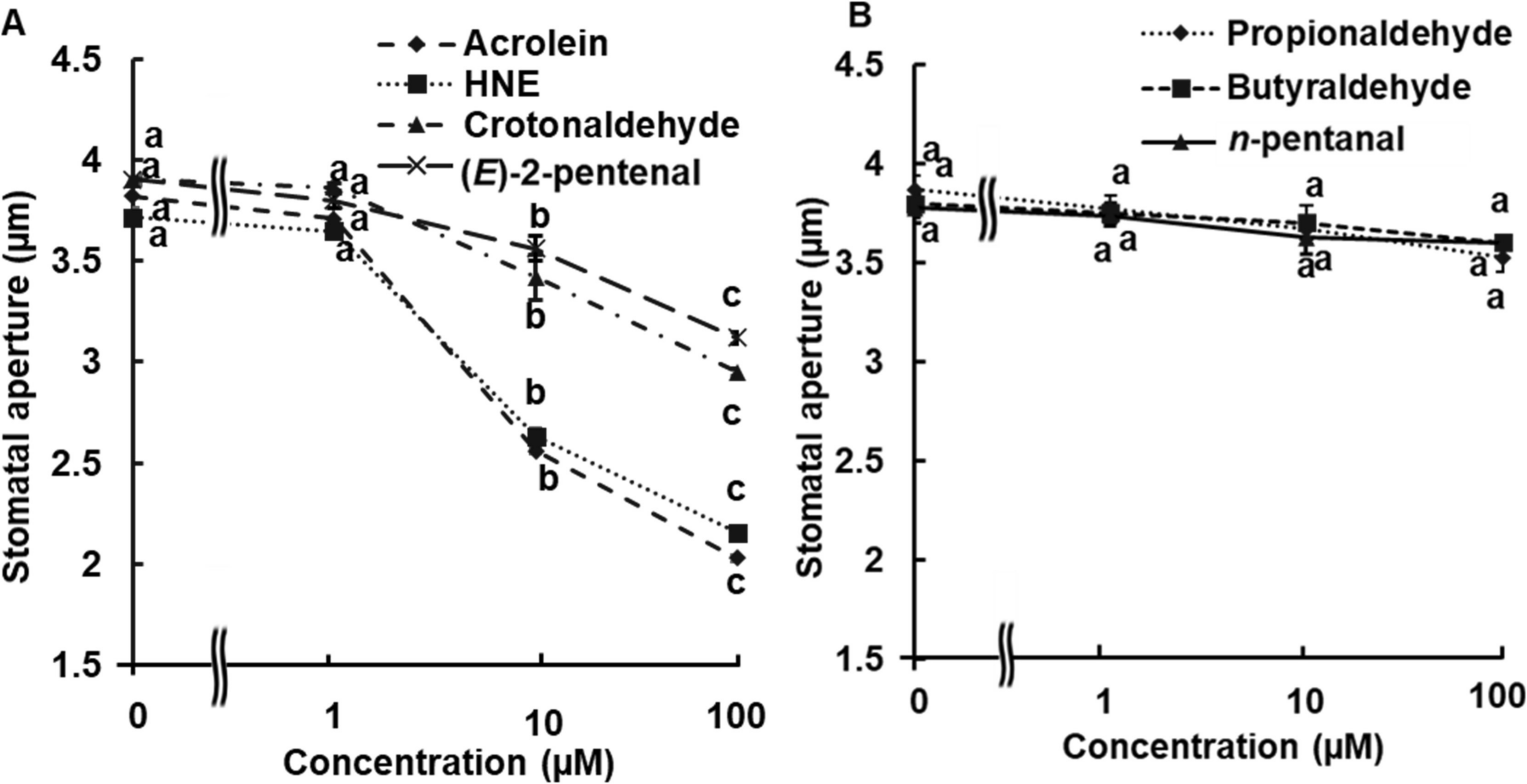
Dose‐dependent effects of RCS and non‐RCS aldehydes on stomatal movement. Arabidopsis leaves were treated with (A) RCS (acrolein, 4‐hydroxy‐(*E*)‐2‐nonenal (HNE), crotonaldehyde, and (*E*)‐2‐pentenal) and (B) non‐RCS aldehydes (propionaldehyde, butyraldehyde, and *n*‐pentanal) under light conditions for 2 h. Each presented data represents the average data of 60 stomata (*n* = 3) ± standard error of the mean. Different letters indicate statistical significance (*p <* 0.05, with Tukey's test).

**FIGURE 6:**
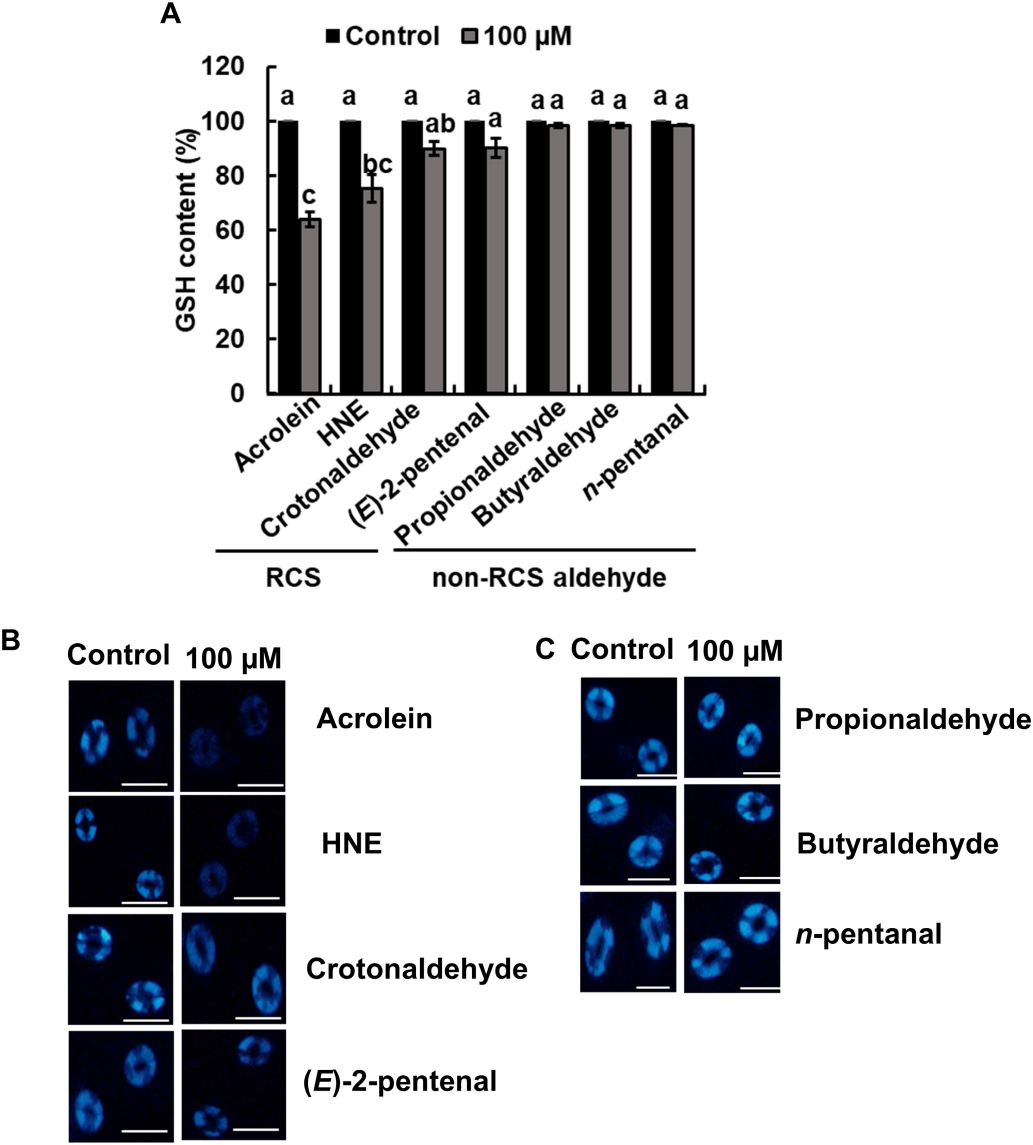
Effects of RCS and non‐RCS aldehydes on GSH content in guard cells. (A) Content of GSH in guard cells treated with RCS (acrolein, 4‐hydroxy‐(*E*)‐2‐nonenal (HNE), crotonaldehyde, and (*E*)‐2‐pentenal) and non‐RCS aldehydes (propionaldehyde, butyraldehyde, and *n*‐pentanal). Epidermal tissues were incubated with 100 μM RCS and non‐RCS aldehydes in the presence of monochlorobimane for 2 h. The vertical axis shows the percentage of GSH content. Data in each bar were gathered from 60 guard cells (*n* = 3) ± standard error of the mean. Different letters indicate statistical significance (*p <* 0.05, with Tukey's test). (B, C) Representative glutathione *S*‐bimane fluorescence images of Arabidopsis guard cells under control, RCS and non‐RCS aldehydes treated conditions. Scale bar: 20 μm.

To further evaluate the involvement of RCS in ITC‐induced stomatal closure, we analyzed dose dependent effects of four major RCS, acrolein, HNE, crotonaldehyde, and (*E*)‐2‐pentenal on stomatal aperture in Arabidopsis at concentrations of 1, 10, and 100 μM (Figure 5A) that were accumulated at larger amounts (Figure 4). The stomatal closure response showed a clear dose dependency, with significant (*p* < 0.05, Tukey's test) stomatal closure observed at 10 and 100 μM for acrolein, HNE, crotonaldehyde, and (*E*)‐2‐pentenal (Figure 5A). Moreover, acrolein and HNE demonstrated stomatal closure more effectively than crotonaldehyde and (*E*)‐2‐pentenal, particularly at higher concentrations (100 μM) (Figure 5A), indicating a differential in efficacy among the various RCS. In contrast, none of the non‐RCS aldehydes tested (propionaldehyde, butyraldehyde, and *n*‐pentanal) significantly elicited stomatal closure (Figure 5B) and GSH depletion (Figure 6) at up to 100 μM in guard cells of Arabidopsis. Moreover, acrolein and HNE at 100 μM decreased the level of GSH (*p* < 0.05, Tukey's test), whereas neither crotonaldehyde nor (*E*)‐2‐pentenal affected the level of GSH in guard cells of Arabidopsis (Figure 6).

### 3.4 Correlation Analysis Among ITC‐Induced Stomatal Closure, GSH Content, ROS Level, and RCS Content

**FIGURE 7:**
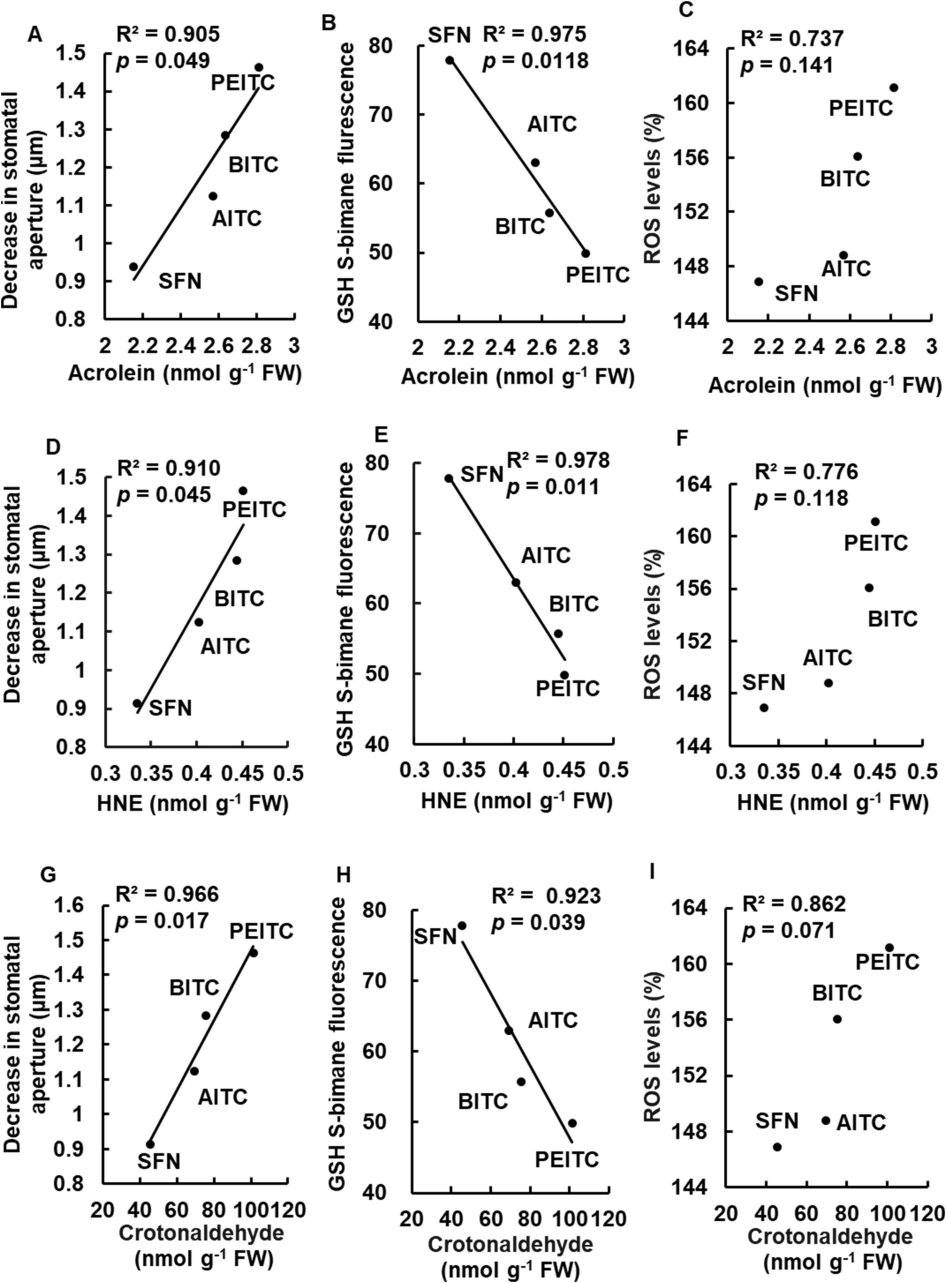
The correlation between two parameters related to ITC‐induced stomatal closure. (A, D, and G) show the decrease in stomatal aperture (μm) versus RCS content in guard cells. (B, E, and H) show GSH content versus RCS content. (C, F, and I) show ROS level versus RCS content. For each correlation, the Pearson correlation coefficient (*r*) and the coefficient of determination (*R* ^2^) are calculated. The statistical significance of each relationship was determined using the *p*‐value, with *p* < 0.05 considered significant. The coefficient of determination (*R* ^2^) is provided to indicate the strength of the linear relationship. Correlation analysis was performed using Microsoft Excel.

Correlation analysis was conducted among decreased stomatal aperture, GSH content, ROS level, and RCS content. A significant positive correlation was observed between the decrease in stomatal aperture and the contents of RCS, acrolein, HNE, and crotonaldehyde in guard cells in response to all four ITCs (*R* ^2^ > 0.90 and *p* < 0.05; Figure 7A,D,G; Table S1). Furthermore, there was a significant negative correlation between the contents of acrolein, HNE, and crotonaldehyde and GSH content for all tested ITCs (*R* ^2^ > 0.92 and *p* < 0.05; Figure 7B,E,H; Table S1). However, there was a positive but not significant correlation between ROS levels and RCS content in guard cells for all four ITCs (*R* ^2^ < 0.89 and *p* > 0.05; Figure 7C,F,I; Table S1).

## 4 Discussion

### 4.1 Reactive Carbonyl Species Mediate ITC‐Induced Stomatal Closure

In this study, ITCs induced stomatal closure (Figure 1), the elevation of ROS levels (Figure 2), and GSH depletion (Figure 3) in guard cells of Arabidopsis, which is consistent with our previous results using Arabidopsis (Afrin et al. 2020; Khokon et al. 2011) and *V. faba* (Sobahan et al. 2015). The RCS scavengers, carnosine and pyridoxamine, inhibit RCS accumulation in abscisic acid‐treated guard cells of Arabidopsis, resulting in inhibition of both GSH depletion and stomatal closure (Islam et al. 2019). In this study, GSH depletion and stomatal closure induced by ITCs (AITC, SFN, BITC, and PEITC) were inhibited by RCS scavengers (Figures 1 and 3), suggesting that RCS is involved in guard‐cell ITC signaling as well as in guard‐cell abscisic acid, methyl jasmonate, and chitosan signaling (Islam et al. 2016, 2019, 2020; Jahan et al. 2025). Application of H_2_O_2_ induces RCS production in Arabidopsis guard cells (Islam et al. 2019), while the elevation of ROS levels by ITCs was not inhibited by RCS scavengers (Figure 2). These results suggest that RCS plays an intermediate role downstream of the elevation of ROS levels in guard‐cell signaling. Furthermore, ITC signaling is likely to share the RCS signal pathway downstream of elevation of ROS level with abscisic acid, methyl jasmonate, and chitosan signaling because the RCS scavengers inhibit abscisic acid‐, methyl jasmonate‐, and chitosan‐induced stomatal closure but not abscisic acid‐, methyl jasmonate‐, and chitosan‐induced elevation of ROS levels (Islam et al. 2019, 2020; Jahan et al. 2025).

The application of ITCs decreased GSH levels to varying degrees (Figure 3). Although it is difficult to discuss the metabolic equilibrium of reactions related to GSH, the different GSH levels can be due to the difference in RCS production (Figure 4), which is dependent on the differences in hydrophobicities/membrane permeabilities and in electrophilicities/reactivities of ITCs. Carnosine and pyridoxamine rescued GSH levels to the statistically same level, although there were variations (Figure 3), suggesting that RCS produced by ITC application is scavenged by carnosine and pyridoxamine. However, the rescued GSH levels did not reach the control level, suggesting that ITCs react with GSH and/or that GSH competes with RCS scavengers in the formation of RCS adducts. Exogenous application of Ca^2+^ chelator, ethylene glycol bis (*β*‐aminoethyl ether)‐*N*,*N*,*N′*,*N′*‐tetraacetic acid (EGTA), and calcium channel blocker, La^3+^, inhibited cytosolic Ca^2+^ elevation induced by AITC in guard cells (Ye et al. 2020). One of the employed RCS scavengers, carnosine, can also chelate Ca^2+^ (Abate et al. 2021). However, the Ca^2+^ chelate formation constant of carnosine is much lower than that of EGTA (Oiki et al. 1994; Abate et al. 2021). Hence, carnosine is not likely to inhibit ITC‐induced stomatal closure through suppression of Ca^2+^ uptake by Ca^2+^ chelation. Furthermore, the possibility that carnosine and pyridoxamine directly interfere with ITC activity was examined. The rate constants of reactions between ITCs (AITC and BITC) and RCS scavengers were much lower than those between RCS (acrolein) and RCS scavengers (Table S2). Although ITC contents in guard cells were not measured, the stronger kinetic preference for the reaction of RCS scavengers with RCS than with ITCs suggests that neither carnosine nor pyridoxamine interferes with ITC activity, supporting our findings that the elevation of ROS levels by ITCs was not inhibited by RCS scavengers (Figure 2).

### 4.2 Reactive Carbonyl Species Contribute to GSH Depletion in Guard Cells

In this study, ITCs (AITC, SFN, BITC, and PEITC) as well as abscisic acid, methyl jasmonate, and chitosan induced RCS production and GSH depletion in Arabidopsis guard cells, which was inhibited by carnosine and pyridoxamine but not completely (Figures 3 and 4; Islam et al. 2019; Islam et al. 2020; Jahan et al. 2025). Moreover, application of acrolein, HNE, crotonaldehyde, and (*E*)‐2‐pentenal induced GSH depletion in the Arabidopsis guard cells (Figure 6; Islam et al. 2019). These results suggest that the produced RCS in response to ITCs is involved in GSH depletion, as a nucleophilic GSH can be depleted through enzymatic or non‐enzymatic conjugation with electrophiles, including RCS (Esterbauer et al. 1975; Zhang et al. 1995). Moreover, Biswas and Mano (2016) demonstrated that RCS reduces GSH levels in tobacco BY‐2 cells, and Davoine et al. (2006) reported that RCS depletes GSH by forming RCS‐GSH conjugates during the hypersensitive response in tobacco. However, there is another possibility that ITCs react with GSH because ITCs are also electrophiles, which can be declined because ITC‐GSH conjugation is not as fast as RCS‐GSH conjugation (Zhang 2001; Sauerland et al. 2021). Furthermore, human GSTs, GSTA1, GSTM1, and GSTP1, and Drosophila GST, GSTD2, were reported to contribute to the formation of ITC‐GSH conjugates (Kolm et al. 1995; Gonzalez et al. 2018). However, it remains to be identified what GST is involved in ITC‐GSH conjugates in guard cells.

Moreover, in contrast to GSH peroxidases in animals, Arabidopsis GSH peroxidases use thioredoxin as an electron donor rather than GSH to scavenge H_2_O_2_ and organic hydroperoxides (Iqbal et al. 2006), suggesting that ROS production is not closely involved in the GSH depletion in guard cells. Taken together, RCS rather than ITCs is likely to be directly related to GSH depletion through RCS‐GSH conjugate formation in guard cells responding to the application of ITCs, although the involvement of GST(s) in this process remains unclear.

### 4.3 Reactive Carbonyl Species, Especially Acrolein and HNE, as Key Regulators of Stomatal Closure

Production of a variety of aldehydes and ketones is induced by not only ITCs (AITC, SFN, BITC, and PEITC) but also abscisic acid, methyl jasmonate, and chitosan (Figure 4; Islam et al. 2016; Islam et al. 2019; Islam et al. 2020; Jahan et al. 2025). The tested RCS (acrolein, HNE, crotonaldehyde, and (*E*)‐2‐pentenal) induced stomatal closure at 10 and 100 μM (Figure 5A) in Arabidopsis, while the tested non‐RCS aldehydes (propionaldehyde, butyraldehyde, and *n*‐pentanal) did not induce stomatal closure at up to 100 μM (Figure 5B). These results indicate that RCS rather than non‐RCS aldehydes is attributed to stomatal closure and are consistent with our previous results that stomatal closure is suppressed by overexpression of 2‐alkenal reductase in tobacco, where 2‐alkenal reductase mediates conversion from RCS to non‐RCS aldehydes (Islam et al. 2016, 2020; Jahan et al. 2025; Mano et al. 2002). Stomatal closure is induced by acrolein at 10 μM but not at 1 μM (Figure 5A), while ITCs at 50 μM induced stomatal closure (Figure 1). Given that the application of acrolein increases intracellular acrolein concentration to the same level in the guard cells, it can be expected that acrolein content in the guard cells treated with ITC at 50 μM is much higher than 1 μM. Hence, increased acrolein content by around 2 nmol g^−1^ FW in the ITC‐treated epidermal tissues can reach 10 μM, which is consistent with our estimate that concentrations of RCS, including acrolein, can regionally reach 100 μM. Plants typically respond to biotic and abiotic stresses through RCS‐mediated programmed cell death, a downstream effect of ROS production (Biswas and Mano 2016). However, Biswas and Mano (2016) showed that 100 μM acrolein caused GSH loss but did not directly induce programmed cell death, as more than 80% of BY‐2 cells remained alive at 5 h. Furthermore, stomatal closure induced by 100 μM acrolein and HNE was reversible, and guard‐cell viability was preserved in Arabidopsis guard cells (Islam et al. 2019). These results suggest that the signaling mechanism of RCS‐induced stomatal closure under our experimental conditions differs from that of programmed cell death.

When guard cells were treated with ITCs, crotonaldehyde was produced 15‐, 100‐, and 6‐times higher than acrolein, HNE, and (*E*)‐2‐pentenal, respectively (Figure 4). However, neither crotonaldehyde nor (*E*)‐2‐pentenal significantly induced GSH depletion (Figure 6A). Furthermore, non‐RCS aldehydes did not induce significant GSH depletion in guard cells (Figure 6A), further supporting the regulatory contribution of RCS to GSH depletion. These results suggest that acrolein and HNE act as key regulatory intermediates in ITC signaling and probably in abscisic acid, methyl jasmonate, and chitosan signaling in guard cells.

Stomatal closure and GSH depletion in guard cells were more strongly induced by exogenous application of acrolein and HNE than that of (*E*)‐2‐pentenal and crotonaldehyde at 10 and 100 μM (Figures 5 and 6). Furthermore, acrolein and HNE are more highly electrophilic than (*E*)‐2‐pentenal and crotonaldehyde (LoPachin and Gavin 2014), which is likely to cause the difference (Figures 5 and 6). The presence of an *α,β*‐unsaturated carbonyl group, as found in acrolein and HNE, enhances their electrophilic reactivity with nucleophilic cellular targets such as thiol groups in GSH, contributing to GSH depletion and downstream stomatal responses (LoPachin and Gavin 2014). Moreover, although the hydrophobicity of a compound can strongly affect its membrane permeability, the difference in activity to induce stomatal closure cannot be due to a difference in hydrophobicity because log *P* values of acrolein, HNE, (*E*)‐2‐pentenal, and crotonaldehyde are 0.11, 1.47, 0.9, and 0.48, respectively. Thus, electrophilicity rather than hydrophobicity of RCS is favorable for RCS to induce stomatal closure and GSH depletion in the guard‐cell ITC signaling pathway.

### 4.4 Correlation Between Signal Components

Correlation analysis revealed a strong positive correlation between the contents of RCS and the decrease in stomatal aperture (Figure 7A,D,G) and a negative correlation with GSH depletion (Figure 7B,E,H). There is no significant correlation between ROS levels and GSH depletion, or between ROS levels and a decrease in stomatal aperture (Afrin et al. 2020). The strong positive correlation between RCS content and the decrease in stomatal aperture, coupled with a negative correlation with GSH depletion (Figure 7), further supports the pivotal role of RCS in modulating stomatal responses to ITCs. Our previous result demonstrated that GSH depletion enhances abscisic acid‐ and methyl jasmonate‐induced stomatal closure without affecting ROS levels (Akter et al. 2012). Hence, RCS contents, rather than ROS levels, are likely to modulate the degree of decrease in stomatal aperture induced by ITCs and are closely related to GSH contents in guard cells, although it remains to be clarified how RCS production level is regulated.

## 5 Conclusion

We can conclude that RCS functions as a signal mediator downstream of ROS production in the Arabidopsis guard‐cell ITC signaling pathway and that acrolein and HNE act as regulatory intermediates to induce stomatal closure.

## Author Contributions

S.F., S.M., and Y.M. designed the research. S.F. performed all experiments with support of M.M.I., S.M., and T.N. I.J. analyzed data with support of M.M.I. T.N., and Y.N. provided suggestions. S.F. and Y.M. wrote the manuscript.

## Funding

This work was supported by the Japan Society for the Promotion of Science (JSPS) KAKENHI (Grants 22H02303 and 25H00921 to Y.M., S.M., and T.N.) and Japan Society for the Promotion of Science (JSPS) Bilateral Collaborations (Grants 120219925 to Y.M., S.M., and T.N.).

## Conflicts of Interest

The authors declare no conflicts of interest.

## Supporting information


**Figure S1:** ppl70775‐sup‐0001‐FigureS1.tif.

**Figure S2:** ppl70775‐sup‐0002‐FigureS2.tif.

**Figure S3:** ppl70775‐sup‐0003‐FigureS3.tif.

**Figure S4:** ppl70775‐sup‐0004‐FigureS4.tif.

**Table S1:** Correlation between two parameters. (A) decrease in stomatal aperture (μm) versus RCS and non‐RCS aldehyde content, (B) GSH content versus RCS and non‐RCS aldehyde content, and (C) ROS levels versus RCS and non‐RCS aldehyde content. For each correlation, the Pearson correlation coefficient (*r*) and the coefficient of determination (R^2^) are calculated. The statistical significance of each relationship was determined using the *p*‐value, with *p* < 0.05 considered significant. The coefficient of determination (*R* ^2^) is provided to indicate the strength of the linear relationship. Correlation analysis was performed using Microsoft Excel.

**Table S2:** Rate constants of reactions between RCS scavengers (carnosine and pyridoxamine) and ITCs (AITC, BITC) and RCS (acrolein). Measurements were performed following the method of Zhang et al. (1995) and Kolm et al. (1995). The reaction rate constant of 50 μM ITCs (AITC and BITC) and 100 μM acrolein with 1 mM carnosine or 0.5 mM pyridoxamine were measured in 100 mM sodium phosphate buffer (pH 6.5).

## Data Availability

The analyzed data sets generated during the study are available from the corresponding author upon reasonable request.
