# Supplementary figures and images for: Reactive Carbonyl Species Mediate Isothiocyanate Signaling Pathway in Arabidopsis thaliana Guard Cells
Source: Physiol Plant. 2026 Feb 11;178(1):e70775. doi: 10.1111/ppl.70775 (PMC12892169; doi:10.1111/ppl.70775)

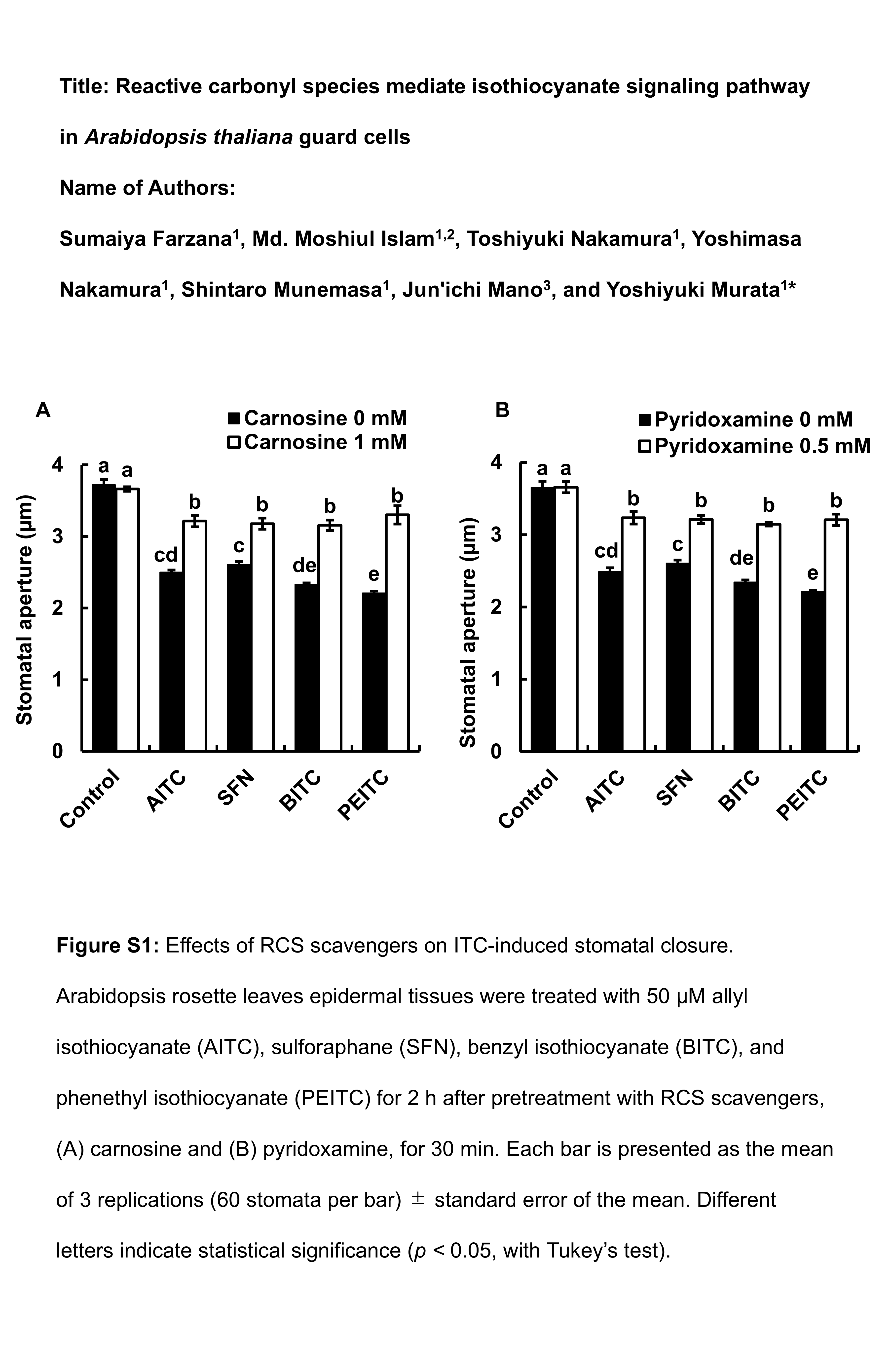

Supplement: Supplementary file 1 — Figure S1: ppl70775‐sup‐0001‐FigureS1.tif. [file PPL-178-e70775-s005.tif]

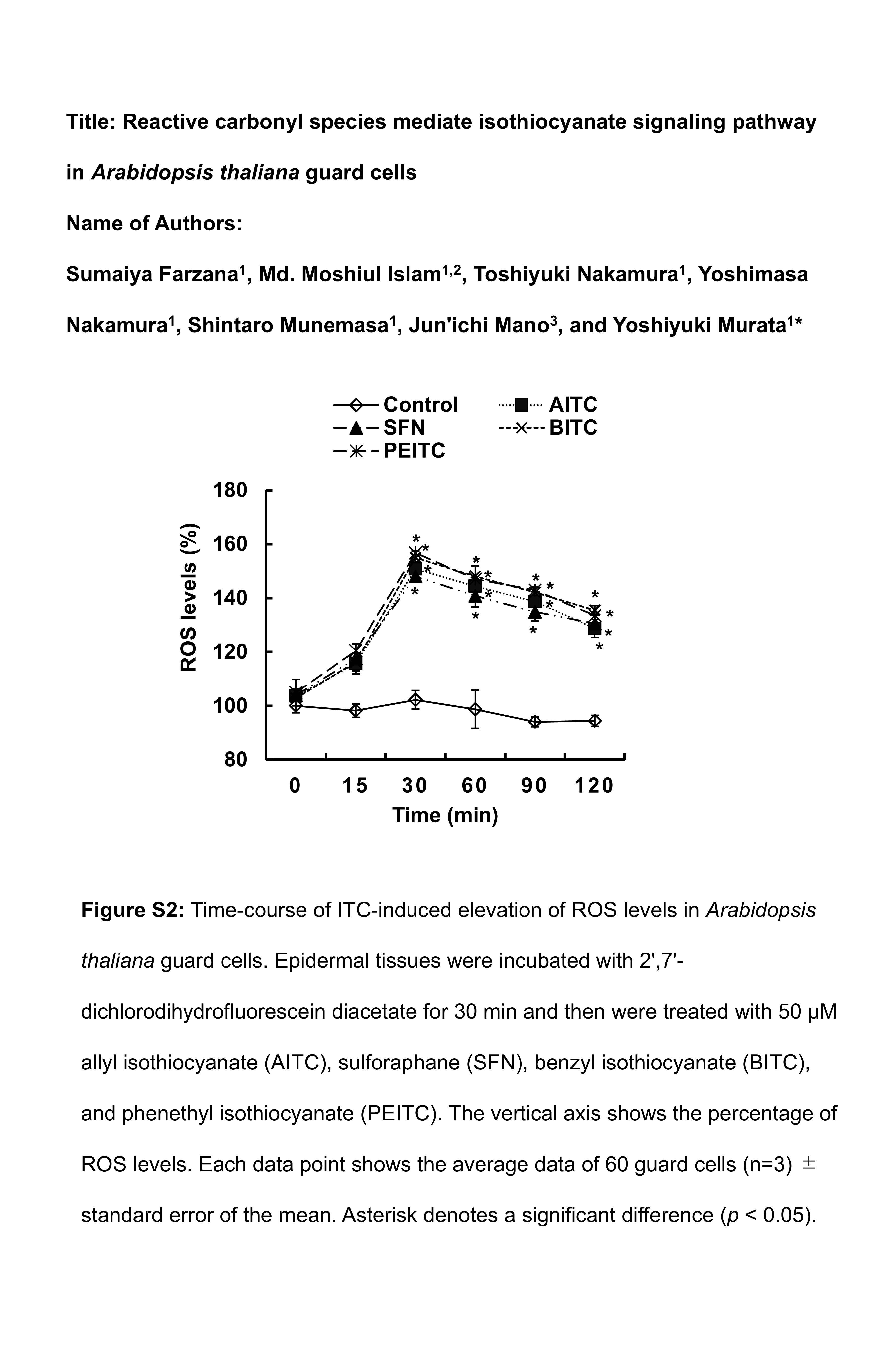

Supplement: Supplementary file 2 — Figure S2: ppl70775‐sup‐0002‐FigureS2.tif. [file PPL-178-e70775-s006.tif]

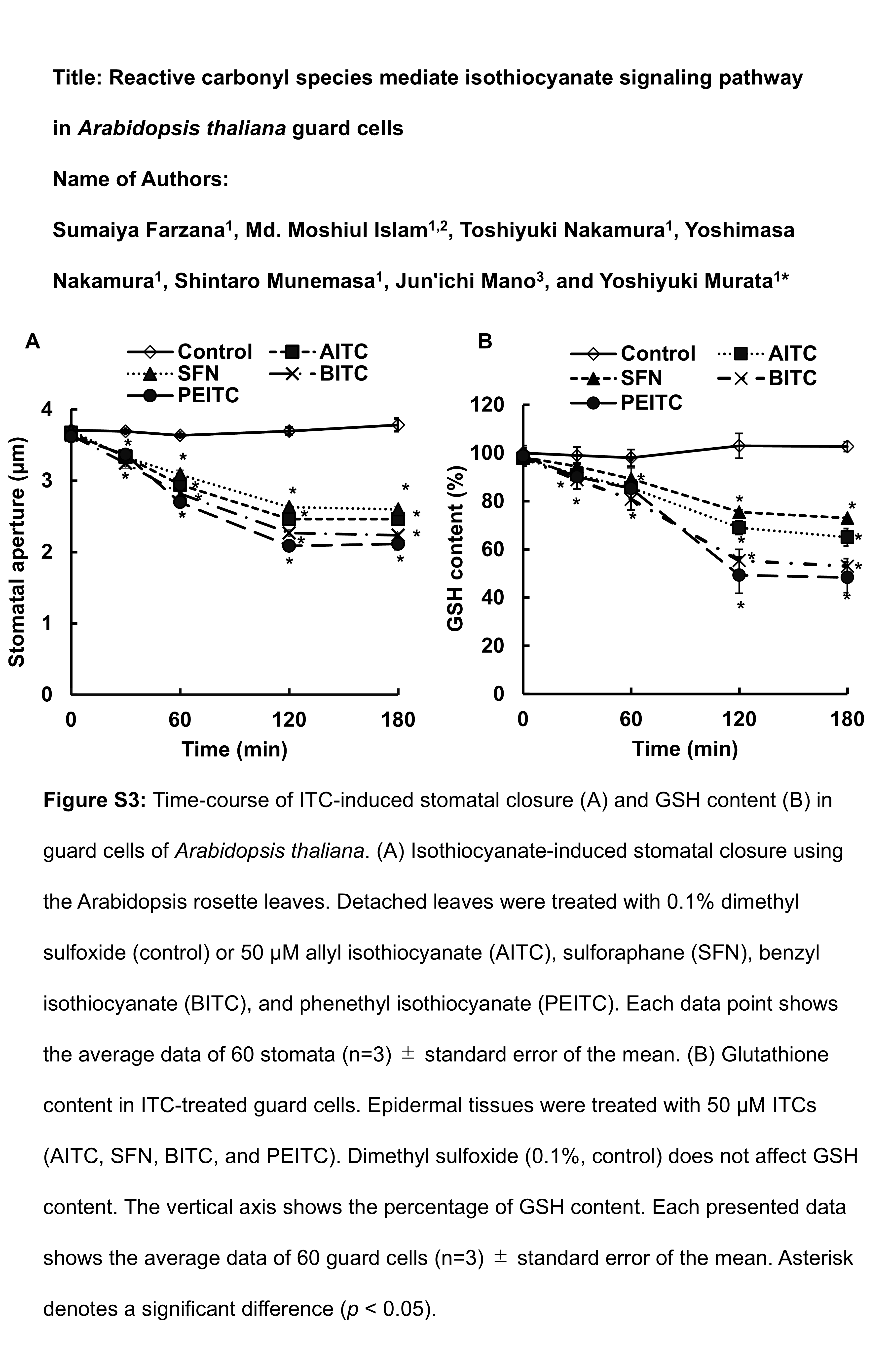

Supplement: Supplementary file 3 — Figure S3: ppl70775‐sup‐0003‐FigureS3.tif. [file PPL-178-e70775-s003.tif]

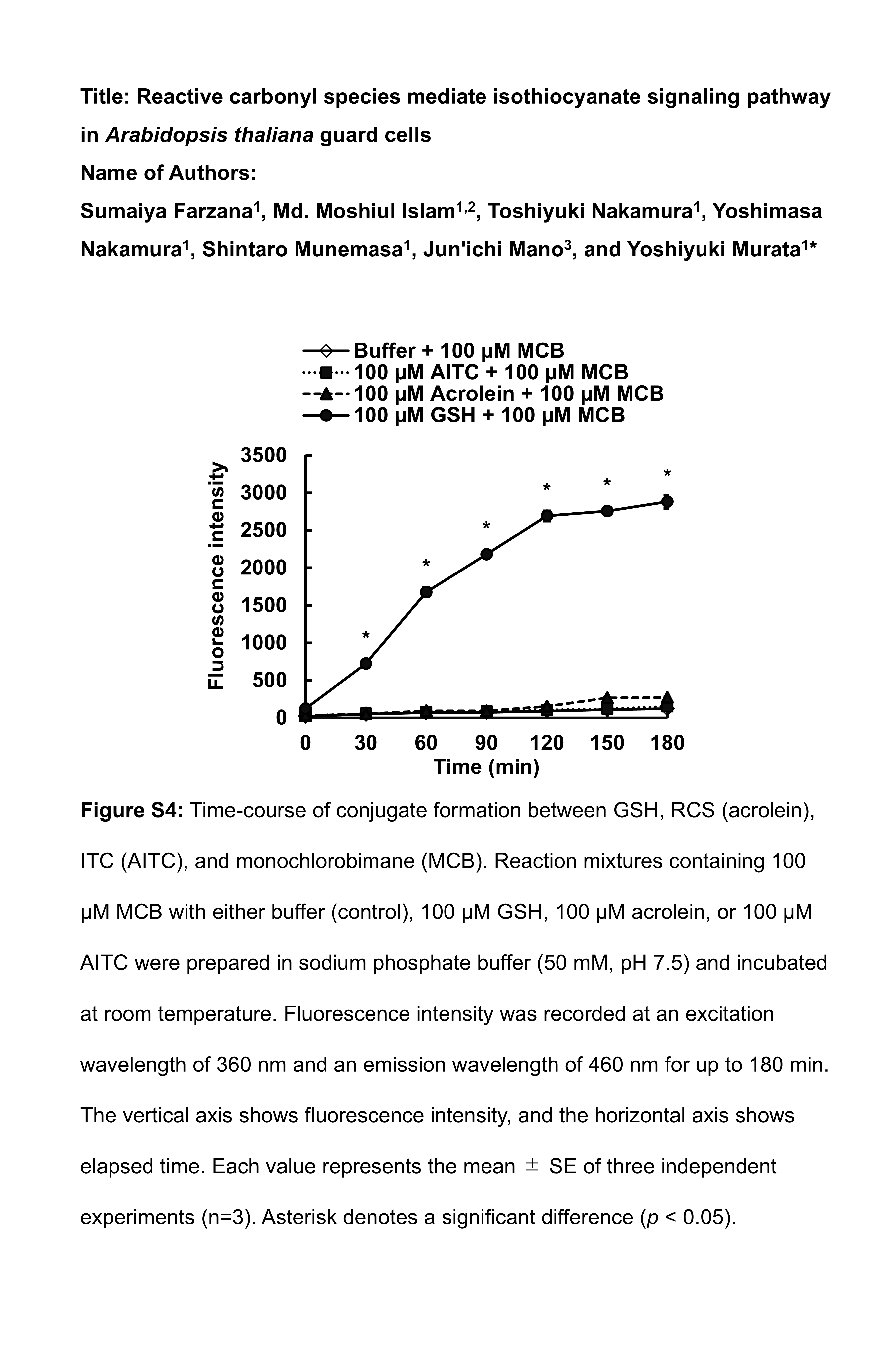

Supplement: Supplementary file 4 — Figure S4: ppl70775‐sup‐0004‐FigureS4.tif. [file PPL-178-e70775-s002.tif]
