# Supplementary material for: Reactive Carbonyl Species Mediate Isothiocyanate Signaling Pathway in Arabidopsis thaliana Guard Cells
Source: Physiol Plant. 2026 Feb 11;178(1):e70775. doi: 10.1111/ppl.70775 (PMC12892169; doi:10.1111/ppl.70775)
Supplement: Supplementary file 5 — Table S1: Correlation between two parameters. (A) decrease in stomatal aperture (μm) versus RCS and non‐RCS aldehyde content, (B) GSH content versus RCS and non‐RCS aldehyde content, and (C) ROS levels versus RCS and non‐RCS aldehyde content. For each correlation, the Pearson correlation coefficient (r) and the coefficient of determination (R2) are calculated. The statistical significance of each relationship was determined using the p‐value, with p < 0.05 considered significant. The coefficient of determination (R 2) is provided to indicate the strength of the linear relationship. Correlation analysis was performed using Microsoft Excel. [file PPL-178-e70775-s004.pdf]

Title: Reactive carbonyl species mediate isothiocyanate signaling pathway in *Arabidopsis thaliana* guard cells

Name of Authors:

Sumaiya Farzana[1], Md. Moshiul Islam[1,2], Toshiyuki Nakamura[1], Yoshimasa Nakamura[1], Shintaro Munemasa[1], Jun'ichi Mano[3], and Yoshiyuki Murata[1*]

Carbonyl species (RCS and non-RCS aldehyde)	(A) Decrease in stomatal aperture (µm) Versus RCS and non-RCS aldehyde content	(B) GSH content Versus RCS and non-RCS aldehyde content	(C) ROS levels Versus RCS and non-RCS aldehyde content
**RCS**			
**HHE**	$R^2 = 0.5937$, $p = 0.229$, r = 0.7705	$R^2 = 0.612$, $p = 0.217$, r = - 0.783	$R^2 = 0.313$, $p = 0.440$, r = 0.559
**(*E*)-2-pentenal**	$R^2 = 0.0025$, $p = 0.950$, r = - 0.049	$R^2 = 0.021$, $p = 0.855$, r = - 0.144	$R^2 = 0.031$, $p = 0.823$, r = - 0.177
**Non-RCS aldehyde**			
**(*Z*)-3-hexenal**	$R^2 = 0.813$, $p = 0.098$, r = 0.901	$R^2 = 0.657$, $p = 0.189$, r = - 0.810	$R^2 = 0.889$, $p = 0.057$, r = 0.942
***n*-hexanal**	$R^2 = 0.109$, $p = 0.669$, r = - 0.330	$R^2 = 0.256$, $p = 0.585$, r = 0.414	$R^2 = 0.219$, $p = 0.468$, r = - 0.531
**Acetaldehyde**	$R^2 = 0.105$, $p = 0.675$, r = 0.324	$R^2 = 0.250$, $p = 0.499$, r = - 0.500	$R^2 = 0.026$, $p = 0.832$, r = 0.162
**Formaldehyde**	$R^2 = 0.324$, $p = 0.430$, r = 0.569	$R^2 = 0.417$, $p = 0.353$, r = - 0.646	$R^2 = 0.095$, $p = 0.691$, r = 0.308
**Propionaldehyde**	$R^2 = 0.620$, $p = 0.212$, r = 0.787	$R^2 = 0.566$, $p = 0.245$, r = - 0.752	$R^2 = 0.770$, $p = 0.119$, r = 0.881
**Butyraldehyde**	$R^2 = 0.115$, $p = 0.660$, r = 0.339	$R^2 = 0.220$, $p = 0.531$, r = - 0.469	$R^2 = 0.071$, $p = 0.785$, r = 0.265

Table S1: Correlation between two parameters. (A) decrease in stomatal aperture (µm)

versus RCS and non-RCS aldehyde content, (B) GSH content versus RCS and non-RCS aldehyde content, and (C) ROS levels versus RCS and non-RCS aldehyde content. For each correlation, the Pearson correlation coefficient (r) and the coefficient of determination (R^2) are calculated. The statistical significance of each relationship was determined using the *p*-value, with $p < 0.05$ considered significant. The coefficient of determination (R^2) is provided to indicate the strength of the linear relationship. Correlation analysis was performed using Microsoft Excel.
