# Supplementary material for: Reactive Carbonyl Species Mediate Isothiocyanate Signaling Pathway in Arabidopsis thaliana Guard Cells
Source: Physiol Plant. 2026 Feb 11;178(1):e70775. doi: 10.1111/ppl.70775 (PMC12892169; doi:10.1111/ppl.70775)
Supplement: Supplementary file 6 — Table S2: Rate constants of reactions between RCS scavengers (carnosine and pyridoxamine) and ITCs (AITC, BITC) and RCS (acrolein). Measurements were performed following the method of Zhang et al. (1995) and Kolm et al. (1995). The reaction rate constant of 50 μM ITCs (AITC and BITC) and 100 μM acrolein with 1 mM carnosine or 0.5 mM pyridoxamine were measured in 100 mM sodium phosphate buffer (pH 6.5). [file PPL-178-e70775-s001.pdf]

Title: Reactive carbonyl species mediate isothiocyanate signaling pathway in *Arabidopsis thaliana* guard cells

Name of Authors:

Sumaiya Farzana[1], Md. Moshiul Islam[1,2], Toshiyuki Nakamura[1], Yoshimasa Nakamura[1], Shintaro Munemasa[1], Jun'ichi Mano[3], and Yoshiyuki Murata[1]*

Compounds	RCS Scavengers	Reaction rate constant, (M^{-1} min^{-1})
**Isothiocyanate (ITC)**		
Allyl Isothiocyanate (AITC)	Carnosine	0.129
Allyl Isothiocyanate (AITC)	Pyridoxamine	0.893
Benzyl Isothiocyanate (BITC)	Carnosine	0.96
Benzyl Isothiocyanate (BITC)	Pyridoxamine	0.092
**Reactive carbonyl species (RCS)**		
Acrolein	Carnosine	63
Acrolein	Pyridoxamine	50

Table S2: Rate constants of reactions between RCS scavengers (carnosine and pyridoxamine) and ITCs (AITC, BITC) and RCS (acrolein). Measurements were performed following the method of Zhang et al. (1995) and Kolm et al. (1995). The reaction rate constant of 50 μM ITCs (AITC and BITC) and 100 μM acrolein with 1 mM carnosine or 0.5 mM pyridoxamine were measured in 100 mM sodium phosphate buffer (pH 6.5).
